# Contributions to Reparative Surgery

**Published:** 1879-02

**Authors:** 


*Contributions to Reparative Surgery;* Showing its application to the treatment of deformities produced by destructive disease or injury; Congenital defects from arrest or excess of development; and Cicatrical Contractions from Burns. By GURDON BUCK, M. D. Illustrated by numerous engravings. New York: D. Appleton & Co. 1876.

This volume of two hundred and thirty-seven pages is devoted to the report of cases with illustrative cuts, occurring in the practice of the Author. The signal achievements of this distinguished Surgeon are apparent from a glance at the illustrations. Surgeons have not experienced more marked triumphs in Reparative Surgery than is here shown in the report of Gurdon Buck, of New York.

